# Efficacy of Transcranial Direct-Current Stimulation in Catatonia: A Review and Case Series

**DOI:** 10.3389/fpsyt.2022.876834

**Published:** 2022-04-27

**Authors:** Alexandre Haroche, Nolwenn Giraud, Fabien Vinckier, Ali Amad, Jonathan Rogers, Mylène Moyal, Laetitia Canivet, Lucie Berkovitch, Raphaël Gaillard, David Attali, Marion Plaze

**Affiliations:** ^1^GHU PARIS Psychiatrie and Neurosciences, site Sainte-Anne, Service Hospitalo-Universitaire, Pôle Hospitalo-Universitaire Paris 15, Paris, France; ^2^Université de Paris, Paris, France; ^3^Department of Neuroimaging, King’s College London, Institute of Psychiatry, Psychology and Neuroscience, London, United Kingdom; ^4^Univ. Lille, Inserm, CHU Lille, U1172 - LilNCog - Lille Neuroscience and Cognition, Lille, France; ^5^Division of Psychiatry, University College London, London, United Kingdom; ^6^South London and Maudsley National Health Service (NHS) Foundation Trust, London, United Kingdom; ^7^Physics for Medicine Paris, Inserm U1273, CNRS UMR 8063, ESPCI Paris, PSL University, Paris, France

**Keywords:** catatonia, transcranial direct-current stimulation, brain stimulation, case series, schizophrenia

## Abstract

Catatonia is a severe neuropsychiatric syndrome, usually treated by benzodiazepines and electroconvulsive therapy. However, therapeutic alternatives are limited, which is particularly critical in situations of treatment resistance or when electroconvulsive therapy is not available. Transcranial direct-current stimulation (tDCS) is a promising non-invasive neuromodulatory technique that has shown efficacy in other psychiatric conditions. We present the largest case series of tDCS use in catatonia, consisting of eight patients in whom tDCS targeting the left dorsolateral prefrontal cortex and temporoparietal junction was employed. We used a General Linear Mixed Model to isolate the effect of tDCS from other confounding factors such as time (spontaneous evolution) or co-prescriptions. The results indicate that tDCS, in addition to symptomatic pharmacotherapies such as lorazepam, seems to effectively reduce catatonic symptoms. These results corroborate a synthesis of five previous case reports of catatonia treated by tDCS in the literature. However, the specific efficacy of tDCS in catatonia remains to be demonstrated in a randomized controlled trial. The development of therapeutic alternatives in catatonia is of paramount importance.

## Significant outcomes

• tDCS has been effective in four cases of catatonia previously described in the literature.
• We hereby present eight cases of catatonia successfully treated by catatonia.
• Further studies are needed to assess tDCS efficacy in catatonia.

## Limitations

• As a case eries, this study includes only eight patients and does not have a control group.
• tDCS effect could be driven by a placebo effect or confounding variables.
• The procedure used (rhythm and number of sessions) was significantly different for each patient.

## Introduction

Although long described as a subtype of schizophrenia, catatonia is a psychomotor syndrome that can now be considered an independent diagnostic entity since the fifth edition of the Diagnostic and Statistical Manual of Mental Disorders (DSM-5) (1). Catatonia includes motor, emotional, and behavioral symptoms which can occur during the course of various psychiatric and neurologic diseases (2). Its clinical manifestations are heterogeneous. According to the DSM-5 (1), the diagnosis of catatonia can be made when three or more of the following twelve clinical features are present: catalepsy, waxy flexibility, stupor, agitation, mutism, negativism, posturing, mannerisms, stereotypies, grimacing, echolalia, and echopraxia. Catatonia is a severe condition with an estimated prevalence of 7.7% in psychiatric inpatient units, according to a recent meta-analysis (3). Among the possible complications of catatonia, malignant catatonia is a rare clinical condition with an estimated mortality of 31% (4).

In addition to etiologic-specific management, there are currently two main treatments for catatonia: benzodiazepines (especially lorazepam) and electroconvulsive therapy (ECT) (5–7). In open trials, 70–80% of patients respond to lorazepam (5). Effectiveness depends on dosage but also on catatonia etiology, with a poorer response rate when catatonia is associated with schizophrenia (8). ECT is currently the key treatment for benzodiazepine-resistant catatonia, with response rates estimated between 53 and 93% (5, 6). Despite a relatively high response rate, there are many limitations to ECT treatment in catatonia. Accessibility to ECT is low (9), which can result in long waiting lists, in addition to the several days often required to perform the baseline work-up. Such a delay to treatment can lead to worsening of symptoms, life-threatening complications or even malignant catatonia. Absolute contraindications to ECT are rare but the requirement for general anesthesia means it can be challenging in patients with severe medical comorbidities. Moreover, chronic catatonia is often ECT-dependent, meaning that catatonic symptoms respond to ECT but relapse when frequency of administration is decreased, or when it is discontinued (5). In summary, available and accessible strategies for the management of catatonia unresponsive to benzodiazepines is an unmet need in psychiatry. A reduction in the duration of untreated catatonia may limit morbidity and mortality, as well as the cost of catatonia through shorter hospitalizations.

Among the alternative treatments that have been investigated over the years (10), non-invasive brain stimulation techniques seem to be a promising approach despite the sparse evidence available. High frequency repetitive transcranial magnetic stimulation (rTMS) has been effective in eight published cases and failed in one case (11, 12). The stimulation target was most frequently the left dorsolateral prefrontal cortex (DLPFC). Besides rTMS, transcranial direct current stimulation (tDCS) relies on the delivery of a low electrical current through two electrodes positioned on the head (anodal excitation and cathodal inhibition). TDCS is a non-invasive and inexpensive neuromodulation tool. It has been reported to be efficient in treating the symptoms of schizophrenia (13) and in other complex neurologic conditions, such as long-lasting disorders of consciousness (14). As with negative symptoms of schizophrenia, decreased DLPFC activity has been associated with catatonia (15–17). Thus, if tDCS applied as in Brunelin’s seminal study and in Valiengo’s study of one hundred patients can improve the negative symptoms of schizophrenia (13, 18), it can be assumed that tDCS applied with the same frontotemporal montage could be effective in catatonia. Indeed, four cases of successful treatment by tDCS in catatonia have been published (11).

*Aims of the study:*

- The first objective of the study is to review the literature on rTMS and tDCS in catatonia.
- The second objective is to assess the effectiveness of tDCS in catatonia through the description of 8 clinical cases.
- The third objective is to assess the tolerance of the use of tDCS in catatonia through the description of 8 clinical cases.

## Materials and Methods

### Literature Search

**FIGURE 1:**
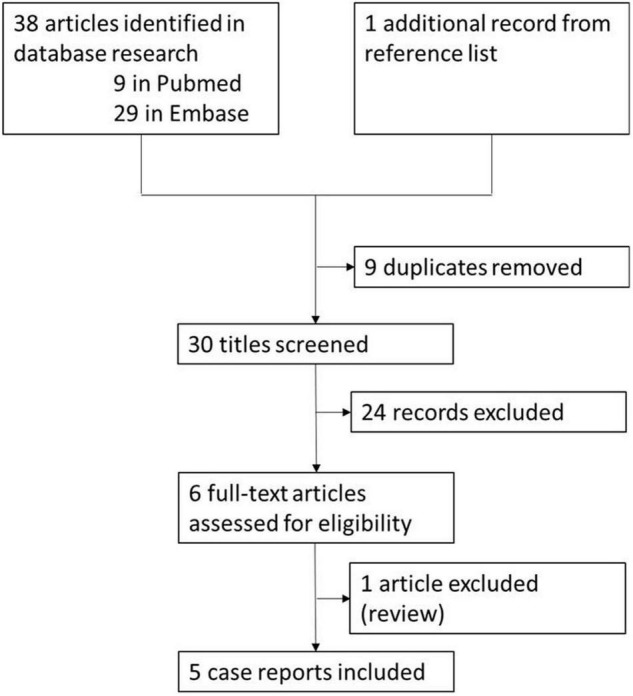
Literature review flowchart.

For the systematic review, we applied the Preferred Reporting Items for Systematic reviews and Meta-Analysis (PRISMA) guideline (19). A systematic literature search on PubMed and Embase was conducted on 30 June 2020 using “catatonic syndrome” (and related terms) and “tDCS” as keywords in order to include case series and case reports related to the topic. We also examined the reference sections from the selected papers to identify any additional relevant studies. Papers were included in the systematic review if (a) they were published in an English-language peer-reviewed journal; (b) the study enrolled patients with catatonia; (c) the study described one or more cases of patients with catatonia treated with tDCS. Article titles and abstracts were screened and excluded from the systematic review for the following reasons: review article; opinion; participants did not have catatonia; tDCS not used. The full text of studies that passed the initial screening was reviewed and potentially excluded based on the same criteria. The study selection process is summarized in the flowchart presented in Figure 1.

### Clinical Study Description

In addition to this systematic review, we report a case series of eight patients with catatonia treated by tDCS. This case series consists of all patients with catatonia treated by tDCS in the department between 2016 and 2020. All patients met criteria for catatonia according to DSM-5 and were hospitalized in a psychiatric ward of GHU Paris Psychiatrie and Neurosciences, Paris, France. All patients were treated unsuccessfully with lorazepam. For seven of them, schizophrenia was the underlying cause of their catatonia. All the patients gave us their consent after clear, fair and adapted information.

### Stimulation Procedures

Placement was guided by the international 10–20 electrode placement system, with the anode over the left DLPFC (midway between F3 and FP1) and the cathode over the left temporoparietal junction (TPJ, midway between T3 and P3). We chose this montage because of promising reports in schizophrenia refractory symptoms (13). Stimulation was performed using a DC-stimulator (Neuroconn) with two 7 × 5 cm sponge electrodes soaked in a 0.9% saline solution. The stimulation level was set at 2 mA for 20 min. The sessions were, as far as possible, conducted twice daily (separated by at least 3 h), on consecutive weekdays. Given the severity of clinical situations and the lack of prior guidelines regarding tDCS in catatonia, the total number of tDCS sessions was not planned *a priori* but guided by daily clinical observation. When tDCS appeared to be efficient, tDCS sessions were continued until complete recovery or until lasting stabilization of the disorder was achieved. TDCS efficacy was assessed by standardized catatonia rating scales: Bush-Francis Catatonia Rating Scale (BFCRS) in 7 cases, and Kanner scale in one case.

### Statistics

For this case series, a statistical analysis of the data was conducted to demonstrate the efficacy of tDCS regardless of the time effect or the effect of concomitantly prescribed drugs. First, we compared the first BFCRS of each patient (before the beginning of tDCS) to the last one (after the course of tDCS) using a paired *t*-test. Then, in order to disentangle the effect of tDCS from other possible factors, we used a general linear mixed model (GLM) approach. BFCRS scores were regressed against two fixed continuous factors, i.e., the cumulative number of tDCS sessions and time (in days) since the first session, and two random factors (intercept and time by patient). In a second model, we included two fixed continuous factors devised to capture the putative effect of benzodiazepines (mainly lorazepam in our sample) and antipsychotics. To do so, a day-by-day proxy for of each drug concentration was computed using a first-order reaction model:


(1) $$C(t)\;=\;C(0)e^{-\;kt}$$

where


(2) $$K=log\left(2\right)*t{}_{1/2}$$

and t_1/2_ is the average half-life of the drug (as reported in the summary of product characteristics, e.g., 15 h for lorazepam). In order to be able to sum different drugs, all benzodiazepine doses were converted to diazepam equivalent (20) and all antipsychotic doses were converted in chlorpromazine equivalent (21). Pharmacokinetics of long-acting injectable antipsychotics is more complex, especially regarding the first injection (22). However, to consider this putative confounding factor, we used an approximation based on time to peak and plasma half-life.

Finally, we tried to isolate a possible acute (symptomatic) effect of tDCS. To do so, we added a last fixed continuous factor indicating the number of sessions performed in the last 7 days (on top of the cumulative number of tDCS sessions since the beginning of the course).

## Results

### Review

In total, five articles (23–27) met the criteria for systematic review (Figure 1). All these manuscripts are case reports of catatonia treated with tDCS. In four cases, tDCS was carried out with the anode positioned over the left DLPFC and the cathode positioned over the right DLPFC. In one case, details about electrode position are not specified. Details of the treatment are provided in Table 1. The number of tDCS sessions varied between 10 (23, 24, 27), 15 (26) and 28 (25). In four cases, tDCS treatment was considered effective in improving catatonic symptoms, while it resulted in no improvement in one case. Symptom reduction varied from 0 to 87%. In two cases, tDCS was conducted after ECT failure (23) or contra-indication (26). In one case, treatment was carried out in a 14-year-old patient (25); the remaining patients were adults.

**TABLE 1:** Published case reports of catatonia treated with direct transcranial direct stimulation (tDCS).

Case/Year	Age/gender	Evolution/malignant features	Underlying diagnosis	tDCS protocol	Results	Symptom reduction
Shiozawa et al. (23)	65/F	Chronic (7 years)/No	Schizophrenia	10 sessions, 1/day, consecutive days, 2 mA, 20 min, anode on left DLPFC, cathode on right DLPFC	Improvement in catatonic symptoms (BFCRS from 32 to 17/69), long lasting (BFCRS = 3/69 after 1 month)	47% then 91%
Costanzo et al. (25)	14/F	Chronic (3 years)/No	Autism spectrum disorder with mild intellectual disability	28 sessions, 1/day, consecutive days, 1 mA, 20 min, anode on left DLPFC, cathode on right DLPFC	Improvement in catatonic symptoms (KCRS from 70 to 28/144), long-lasting (KCRS = 40/144 after 1 month)	60% then 43%
Baldinger-Melich et al. (27)	42/M	Chronic (since adolescence)/Yes (hyperthermia)	Schizophrenia	10 sessions, 1/day, consecutive days, 2mA, 20 min. No information about electrode position	No improvement in catatonic symptoms (BFCRS = 37)	0%
Chen et al. (24)	40/F	Chronic (several months)/No	Schizophrenia	10 sessions, 1/day, consecutive days, 2 mA, 20 min, anode on left DLPFC, cathode on right DLPFC	Improvement in catatonic symptoms (BFCRS from 7 to 3/69) and motor function, not long-lasting	57%
Wysokiński (26)	58/F	Acute/No	Schizophrenia	15 sessions, 1/day: week 1, 1-week interval, week 3, 3-week interval, and week 7. Anode on left DLPCF, cathode on right DLPFC	Improvement of catatonic symptoms (BFCRS from 11 after ECT and before first tDCS course to 2 after the third tDCS course)	81%

*DLPFC, Dorsolateral prefrontal cortex; TPJ, Temporoparietal junction; BFCRS, Bush Francis Catatonia Rating Scale; KCRS, Kanner Catatonia Rating Scale.*

### Case Series

**FIGURE 2:**
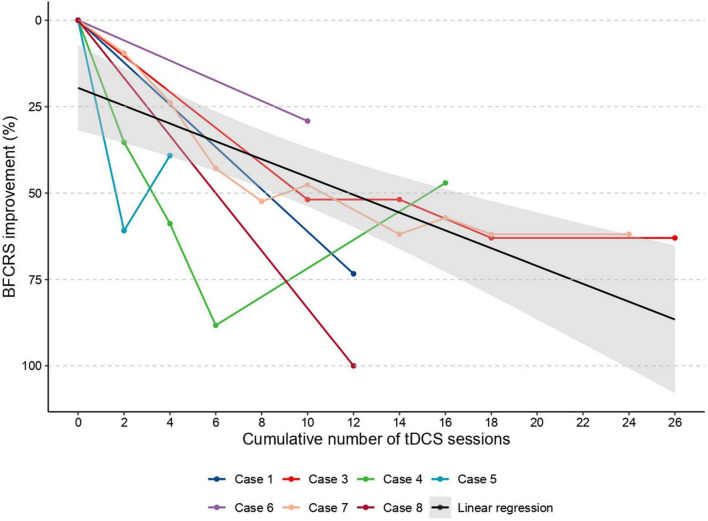
Individual data about the evolution of BFCRS score according to the cumulative number of tDCS sessions. Case 2, for whom catatonia severity was assessed using Kanner scale, is not shown in this figure. In black, the regression line of the scatter plot.

In all our 8 cases, tDCS was associated with an improvement in catatonic symptoms. Patient and tDCS treatment characteristics are available in Table 2. A full description of cases is available in Supplementary Material. The mean age of subjects was 33 years old (SD 6.8), and there was an equal number of men and women. A significant reduction in catatonic symptoms (between 29 and 100%) was observed in all patients. The number of tDCS sessions performed for each patient ranged from 5 to 34 sessions. The tDCS sessions were well tolerated, the only adverse events reported being a burning sensation or tingling. tDCS was effective in three cases in which ECT was poorly tolerated or contra-indicated (cases 1, 2, and 7). In two cases (cases 4 and 8), tDCS helped to avoid ECT treatment. In two cases (cases 3 and 7), catatonic symptoms worsened after tDCS cessation, and improved after tDCS re-challenge. Data about relative evolution of BFCRS scores for each patient are summarized in Figure 2. Clinical details about each case are available in Supplementary Material.

**TABLE 2:** Case series of catatonia treated with transcranial direct stimulation.

Case/Year	Age/Gender	Evolution/Malignant features	Underlying diagnosis	tDCS protocol	Results	Symptom reduction
Case 1/2016	24/F	Acute/no	Schizoaffective disorder	12 sessions, 2/day, consecutive days, 2 mA, 20 min, anode on left DLPFC, cathode on left TPJ	Improvement in catatonic symptoms (BFCRS from 15 to 4/69), long-lasting	73%
Case 2/2016	25/M	Acute/yes : hyperthermia, tachycardia	Schizophrenia	20 sessions, 2/day, consecutive days, 2 mA, 20 min, anode on left DLPFC, cathode on left TPJ	Improvement in catatonic symptoms (KCRS from 68 to 14/144)	79%
Case 3/2019	54/M	Chronic (several months)/no	Schizophrenia and Autism Spectrum Disorder	16 sessions, 2/day, consecutive weekdays, 2 mA, 20 min, anode on left DLPFC, cathode on left TPJ. Then consolidation sessions 2/day every 15 days	Improvement in catatonic symptoms (BFCRS from 27 to 13/69) then long-lasting. Improvement in cognitive and hallucinatory symptoms	52%
Case 4/2019	58/F	Acute/yes : tachycardia	Schizophrenia	20 sessions, 2/day, consecutive weekdays, 2 mA, 20 min, anode on left DLPFC, cathode on left TPJ	Improvement in catatonic symptoms including tachycardia (BFCRS from 17 to 9/69). Improvement of hallucinatory symptoms.	47%
Case 5/2019	59/F	Acute/no	Schizophrenia	5 sessions, 2–3/day, consecutive weekdays, 2 mA, 20 min, anode on left DLPFC, cathode on left TPJ	Improvement in catatonic symptoms (BFCRS from 23 to 9/69)	61%
Case 6/2019	26/M	Chronic (several months)/no	Schizophrenia and Autism Spectrum Disorder	10 sessions, 2/day, consecutive weekdays, 2 mA, 20 min, anode on left DLPFC, cathode on left TPJ	Improvement in catatonic and hallucinatory symptoms (BFCRS from 24 to 17/69 2-months later)	29%
Case 7/2019	54/M	Chronic (several months)/no	Schizophrenia	34 sessions, 2/day, consecutive weekdays, 2 mA, 20 min, anode on left DLPFC, cathode on left TPJ	Improvement in catatonic symptoms (BFCRS from 22 to 8/69), relapse when stopped.	64%
Case 8/2019	24/F	Acute/yes : excessive sweating	Bipolar Disorder	14 sessions, 2/day, consecutive weekdays, 2 mA, 20 min, anode on left DLPFC, cathode on left TPJ	Remission of catatonic symptoms (BFCRS from 14 to 0/69)	100%

*DLPFC, Dorsolateral prefrontal cortex; TPJ, Temporoparietal junction; BFCRS, Bush-Francis Catatonia Rating Scale; KCRS, Kanner Catatonia Rating Scale.*

**FIGURE 3:**
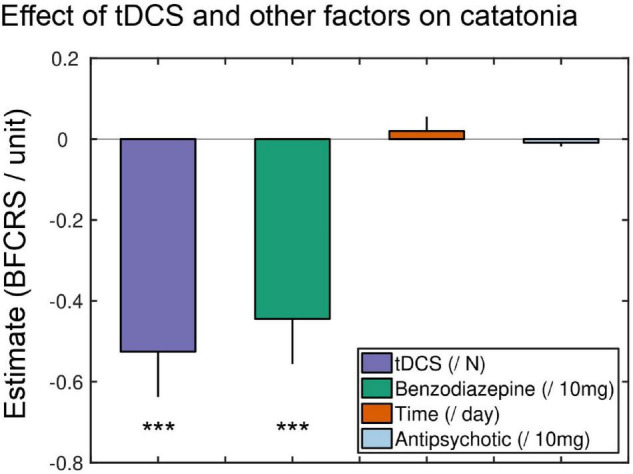
Effect of tDCS and other factors on BFCRS. Coefficient estimates of the general linear mixed model. TDCS regressor was expressed in number of sessions since the beginning of the course, meaning that each tDCS session resulted in a 0.45 decrease of BFCRS score. Time was expressed in days. For benzodiazepine and antipsychotic a proxy for concentration was computed (see section “Materials and Methods”). For comparability of estimate size, benzodiazepine regressor was expressed in centigram (10 mg) of diazepam equivalent while antipsychotic regressor was expressed centigram of chlorpromazine equivalent. Error bars represent standard errors of coefficient estimates. ***Statistically significant.

Despite the small number of patients, the difference between first and last BFRCS score was highly significant [*t*(6) = 5.2, *p* = 0.002, *n* = 7, exclusion of the patient with Kanner scale], indicating that catatonia was more severe at the beginning [16.4 (SD 4.1)] than at the end of the course of tDCS [8.9 (SD 6.5)]. Similarly, the first linear mixed model, including only time as a possible confounding factor, revealed a significant effect of the cumulative number of tDCS sessions (estimate = –0.46/session; *p* < 0.001), but no effect of time (*p* = 0.70). However, all patients received many medications on top of tDCS. To isolate the effect of tDCS from the effect of other treatments (and especially lorazepam, which is the most used drug in catatonia), we included two other possible confounding factors aiming to capture the effect of benzodiazepines and antipsychotic drugs (Figure 3). As predicted, we observed an inverse relation between the (estimated) concentration of benzodiazepines (estimate = –0.45/10 mg of diazepam equivalent; *p* < 0.001) and BFCRS scores, but no effect of antipsychotics (*p* = 0.35). However, and critically, the effect of tDCS was still highly significant (estimate = –0.53 / session; *p* < 0.001). Finally, we aimed to disentangle an acute effect of tDCS from its long-term cumulative effect. To do so, we also included the number of tDCS sessions performed in the last 7 days in the model in addition to the cumulative number of sessions since the beginning of the course. Critically, the number of tDCS sessions performed in the previous 7 days and the cumulative number of sessions performed since the beginning of the treatment were both significant (estimate = -0.57/session; *p* = 0.003 and estimate = -0.39/session; *p* < 0.001), on top of the effect of benzodiazepines (*p* = 0.035). Overall, these results indicate that tDCS seems to be effective in reducing catatonic symptoms over and above symptomatic drugs such as lorazepam, both in the short and long term.

## Discussion

In this paper, we reviewed the five previously published case reports evaluating the effect of tDCS in catatonia, and we reported a new case series of eight patients, which constitutes to the best of our knowledge the largest series published so far. In our case series, we observed a rapid decrease in BFCRS in seven patients. Moreover, we used a mixed model approach to isolate the effect of tDCS from other confounding factors such as time (spontaneous evolution) or co-prescriptions. Interestingly, we observed an acute effect of the number of tDCS sessions performed in the last 7 days on top of a long-term cumulative number of sessions since the beginning of the course. Although very preliminary, this proof-of-concept study suggests that tDCS may be a promising avenue in the management of this severe and life-threatening condition.

### Study Population

When we merged our case series with previously published cases (Table 1), the mean age was 42 years with a sex-ratio of 8 females/5 males. The underlying etiology of the catatonia was schizophrenia (or a disorder from the schizophrenia spectrum) in 11 out of 13 patients. Current catatonic episode was chronic in 7 out of 13 patients. Importantly, these two factors are classically associated with poor prognosis (8, 28). Moreover, the mean BFCRS was 16 (SD 4.1), and malignant signs were observed in 3 patients. Overall, while patients described in the present paper were very heterogeneous, both in terms of clinical presentation and underlying pathologies, the average severity was high. The number of tDCS sessions was extremely variable, from 5 (Case 5) to 34 (case 7). In most patients, a clinically relevant improvement of catatonic symptoms was observed. Importantly, in at least two cases (cases 3 and 7), a challenge-rechallenge effect was observed, with catatonic symptoms worsening after tDCS discontinuation and improving again after tDCS resumption. This finding was confirmed by the GLM we conducted, in which we considered the effect of time. Finally, tDCS was generally well tolerated, the only adverse events reported being a burning sensation or tingling.

### Mechanism of Action of Transcranial Direct-Current Stimulation Treatment in Catatonia

Obviously, we can only speculate on the putative mechanisms underlying the effect of tDCS on catatonia. Clinical observations, anatomopathological and brain imaging studies allow us to propose explanatory models of catatonia based also on our knowledge of the physiological functioning of emotional processing and movement. Benefic effect of benzodiazepines and NMDA antagonists, and negative effect of neuroleptics in catatonia, suggest a role of GABAergic, glutamatergic and dopaminergic pathways in catatonia pathophysiology (5, 10). Dysfunction of frontal cortex appears to be central to the pathophysiology of catatonia, as suggested by Northoff’s studies showing that catatonia is related to GABAergic orbitofrontal deficits following negative emotional processing, highlighting a key role for the overloading of fear regulation by orbitofrontal cortex in the development of catatonia (15, 29, 30). The motor anosognosia characterizing catatonia is supposed to be linked to a dysfunction of the dorsolateral prefrontal cortex as well as the parietal cortex (29). Other recent studies also point to the role of the supplementary motor area (31). More broadly, catatonia is thought to be related to dysconnectivity between different cortical regions: orbitofrontal, prefrontal, motor, and parietal areas; but catatonia also involved dysfunction of cortico-subcortical loops. Thus, Fricchione and Beach proposes a model based on abnormalities in the regulation of the opening and closing of the thalamic filter to explain hypokinetic or hyperkinetic catatonia (32). It is probably a more global dysfunction, characterized by a dysconnectivity of networks involving different cortical areas and different cortico-subcortical loops involved in cognitive, emotional and motor processing, that can account for the major alteration of the subject’s ability to properly interact with their environment (32, 33).

Regarding the mechanism of action of tDCS, it is thought to have a short-term local action related to the change in excitability of stimulated cortical neurons but also a more diffuse and delayed one, involving dopaminergic, glutamatergic and GABAergic systems and resulting in modulation of regional brain activity and functional connectivity (34, 35). At the neurobiological level, the recent demonstration that tDCS increases dopamine release in the ventral striatum of healthy subjects (36) is particularly interesting, and could be a possible explanation of the effectiveness of tDCS in catatonia. At the neural level, the effect of tDCS on catatonia may be mediated by an increase in DLPFC activity, which had been suggested to be the crossroads between the horizontal (cortico-cortical relation) and vertical modulations (cortical-basal ganglia relation) involved, respectively, in emotional/behavioral and motor symptoms of catatonia (29). In the four patients previously reported in the literature, the anode was positioned over the left DLPFC and the cathode over the right DLPFC. Conversely, in our case series, the anode was positioned over the left DLPFC (midway between F3 and FP1) and the cathode over left temporo-parietal junction (TPJ, midway between T3 and P3). The same montage was previously used to alleviate hallucinations (13, 37, 38) and negative symptoms in schizophrenia (18). Furthermore, it is interesting to note that tDCS is effective in reducing motor prediction errors in patients with schizophrenia (39) as motor prediction errors are a hypothesis of the underlying mechanism of catatonia (32, 40).

Finally, tDCS could have a positive effect on the functional dysconnectivity suspected in catatonia (33). As far as dysconnectivity is concerned, a recent paper investigated the effect of tDCS in a large cohort of patients with long-lasting disorders of consciousness (14). Critically, responders showed increases of power and long-range cortico-cortical functional connectivity in the theta-alpha band and a larger and more sustained P300, suggesting improved conscious access to auditory novelty. To what extent catatonia—or at the very least some catatonias—should be considered as a disorder of consciousness is an open issue, as signatures of consciousness have not been investigated in catatonia so far. Nevertheless, the demonstration that tDCS may increase long range cortico-cortical functional connectivity in severe brain injuries suggests that such a mechanism could underlie tDCS therapeutic effects in other functional dysconnectivity disorders, such as catatonia.

Due to the observational nature of this study, we cannot exclude that improvement in catatonia features was driven—at least in some patients—by a placebo effect and/or confounding variables. Indeed, the placebo effect may be particularly important with medical devices and all patients received various medication and interventions that are likely to interact with tDCS (41). Similarly, the procedure was tuned and adjusted specifically for each patient, meaning that the number and the rhythm of sessions were not homogeneous in our case series. Should its efficiency be confirmed, the optimal frequency of tDCS sessions is likely to be an important question. As a matter of fact, recent papers showed that the effect of intermittent theta-burst stimulation (iTBS) in depression could be dramatically increased with multiple sessions per day at optimally spaced intervals (42). However, careful designs may be required to disentangle the acute effect of tDCS from its long-term cumulative effect.

### Future Research

In the future, a randomized clinical trial using sham tDCS will be needed to properly assess the efficacy of tDCS in catatonia. Another important issue will be to what extent tDCS might be considered as a specific treatment of catatonia, independent from underlying etiology, or if at least part of its effect may be mediated by its action on whichever disorder underlies catatonia. Indeed, as already mentioned, the montage we used was shown to be effective in alleviating the positive but also negative symptoms of schizophrenia (13, 18), and most of our patients had schizophrenia or a schizophrenia spectrum disorder. However, it is worth noting that in our case series, the only case where we found a complete improvement in BFCRS (100%) was also the only case where bipolar disorder was the underlying cause. This implies that a trial evaluating the efficacy of tDCS should be conducted not only in catatonic patients with schizophrenia spectrum disorders but also in catatonic patients with mood disorders. In addition, the study of other montages, and other targets [such as cerebellum (43)], rhythm or length of treatment could be interesting to determine the specificity of our montage in the treatment of catatonia. In a trial, it might be useful to stratify randomization by duration of catatonia to account for the heterogeneity of this disorder. Furthermore, the treatment protocol could be adapted to the symptoms of each patient.

## Conclusion

tDCS could revolutionize catatonia management. Indeed, this non-invasive, inexpensive, easily implemented neuromodulatory tool could be used (1) instead of ECT for tDCS-responding patients, thus improving ECT availability for resistant patients; (2) while awaiting ECT, thus reducing life-threatening complications that can occur in the time-course of catatonia; or even (3) as a long-term strategy for ECT-dependent catatonia, thus alleviating the burden of chronic catatonia.

## Data Availability Statement

The original contributions presented in the study are included in the article/Supplementary Material, further inquiries can be directed to the corresponding author/s.

## Ethics Statement

Ethical review and approval was not required for the study on human participants in accordance with the local legislation and institutional requirements. Written informed consent for participation was not required for this study in accordance with the national legislation and the institutional requirements.

## Author Contributions

All authors listed have made a substantial, direct, and intellectual contribution to the work, and approved it for publication.

## Conflict of Interest

JR has held an advisory meeting with representatives from Promentis Pharmaceuticals, Inc. regarding drug development in an unpaid capacity. FV been invited to scientific meetings, consulted and/or served as speaker and received compensation by Lundbeck, Servier, Recordati, Janssen, Otsuka, Chiesi, and LivaNova. RG has received compensation as a member of the scientific advisory board of Janssen, Lundbeck, Roche, SOBI, Takeda. He has served as consultant and/or speaker for Astra Zeneca, Boehringer-Ingelheim, Pierre Fabre, Lilly, Lundbeck, LVMH, MAPREG, Novartis, Otsuka, Pileje, SANOFI, Servier and received compensation, and he has received research support from Servier. MP has served as speaker and received compensation by Lundbeck, Janssen, and LivaNova. The remaining authors declare that the research was conducted in the absence of any commercial or financial relationships that could be construed as a potential conflict of interest.

## Publisher’s Note

All claims expressed in this article are solely those of the authors and do not necessarily represent those of their affiliated organizations, or those of the publisher, the editors and the reviewers. Any product that may be evaluated in this article, or claim that may be made by its manufacturer, is not guaranteed or endorsed by the publisher.
