# Supplementary material for: Efficacy of Transcranial Direct-Current Stimulation in Catatonia: A Review and Case Series
Source: Front Psychiatry. 2022 Apr 27;13:876834. doi: 10.3389/fpsyt.2022.876834 (PMC9093033; doi:10.3389/fpsyt.2022.876834)
Supplement: Supplementary file 1 [file Data_Sheet_1.doc]

**Supplementary materials**

**Description of the 8 patients**

Case 1 (2016)

Mrs. B was a 24-year-old female with schizoaffective disorder who was first hospitalized in 2015 for melancholic depression with psychotic and catatonic symptoms. Back then, catatonic syndrome was successfully treated with lorazepam up to 3 mg per day. As she presented venlafaxine-induced mania, she received 14 sessions of ECT, and was then stabilized by lithium and aripiprazole. A few months later, she stopped aripiprazole and swiftly developed catatonic symptoms including perplexity, stupor, posturing, staring, associated with melancholic symptoms such as intense guilt. Complementary examinations, including electroencephalogram (EEG), brain magnetic resonance imaging (MRI), and complete blood count, returned normal. Lorazepam was introduced and progressively increased up to 15 mg/day, without efficacy. ECT was initiated but from the very first session, catatonic symptoms worsened with the emergence of agitation, negativism, rigidity and combativeness. ECT had to be be stopped after 10 sessions because of poor neurological tolerance and insufficient efficacy. Quetiapine was then carefully introduced, up to 800 mg/day. tDCS was initiated with the first session performed on March 11, 2016, 7 days after the last session of ECT. Twelve sessions of tDCS were performed (twice a day for 6 consecutive days), concomitantly with the progressive increase in the dosage of quetiapine. A rapid reduction in catatonic symptoms was observed. The rest of the treatment consisted of lorazepam in doses ranging from 2.5 to 7.5 mg/day. BFCRS was scored retrospectively from medical observations and went from 15/69 on March 11 (before tDCS) to 4/69 on May 9 (after tDCS), meaning an overall 73% symptom reduction. Nevertheless, delusion, disorganization and mood disturbance were still observed. With clozapine (400 mg/day) and aripiprazole (30 mg/day) reintroduced on top of lithium (600 mg/day), remission was achieved and the patient was discharged from hospital.

Case 2 (2016)

Mr. H was a 25-year-old male patient with schizophrenia. Between 2014 and 2016, he received several oral antipsychotics including aripiprazole, olanzapine and risperidone. In 2016, he was hospitalized for an exacerbation of psychotic symptoms with disorganization, delusion and hallucinations, as a result of risperidone discontinuation. Risperidone was reintroduced, without efficacity, and haloperidol injections were initiated. The day after the first injection, the patient became stuporous, mute, staring, grimacing, refusing to eat and drink. Kanner Scale was rated at 68/144. Beyond these catatonic symptoms, fever, tachycardia, and elevation of creatine kinase and C-reactive protein were observed. A neuroleptic malignant syndrome was suspected and emergency imaging and biological explorations were carried out (lumbar puncture, cerebral and thoracic computed tomography scan). A bilateral pulmonary embolism was diagnosed and Mr H was transferred to intensive care unit (ICU). However, several days later, catatonic symptoms and fever were still observed. ECT was initiated in ICU resulting in an improvement after the very first session (resolution of mutism and negativism, resumption of oral intake). Unfortunately, a cardiac arrest occurred during the 5th ECT session, resulting in further ECT contra-indication. After a positive zolpidem test, lorazepam was titrated up to 17.5 mg/day, without any improvement. tDCS was initiated with the first session performed a few days after the last ECT session, while the patient was still in ICU. A rapid improvement in catatonic symptoms was observed. The patient was discharged from ICU and transferred to a psychiatric unit. In total, 20 sessions of tDCS were performed (twice a day, 6 consecutive days) with further reduction of catatonic symptoms. Kanner score went from 68 before tDCS to 14/144 after tDCS, i.e. with an overall 79% symptom reduction. Depressive mood, inappropriate guilt and hallucinations were still observed so quetiapine was cautiously introduced, resulting in symptomatic relief without relapse of catatonic syndrome.

Case 3 (2019)

Mr. D was a 54-year-old male patient with autism spectrum disorder and schizophrenia. During childhood, he received valproate for 5 years for epilepsy which was latter discontinued after several normal EEGs. Between 2001 and 2017, he was treated by risperidone 1 mg/day. Since 2017, he suffered from a severe major depressive episode with catatonic syndrome, which was first treated on an outpatient basis. Risperidone 1 mg/day was discontinued and olanzapine 10 mg was initiated. After a positive zolpidem test, lorazepam (up to 7.5mg/day) and sertraline (150 mg/day) were initiated, and olanzapine was switched to quetiapine (300 mg/day). After 3 weeks of treatment, hallucinations, persecutory delusions and catatonic symptoms were still observed and the patient was hospitalized. At arrival, fixed gaze, rigidity, negativism, echolalia, stereotypy and perseveration were observed, with a BFCRS score of 27/69. EEG, MRI and lumbar puncture returned normal. tDCS was initiated with the first session performed the 5^th^ of February 2019. Sixteen sessions of tDCS were performed (twice a day for 8 consecutive weekdays) resulting in a rapid reduction in catatonic symptoms. BFCRS went from 27/69 on February 4 (before tDCS) to 13/69 on February 15, i.e. with an overall 52% symptom reduction. Given catatonic syndrome improvement, together with cognitive disorders and hallucinations improvement, consolidation sessions were decided (2 sessions every 2 weeks). This strategy resulted in long-term clinical stability. Since then, because of psychiatric symptoms worsening during the spacing or discontinuation of tDCS, the frequency of sessions is maintained to two sessions every 2 weeks.

Case 4 (2019)

Mrs. M was a 58-year-old female patient with schizophrenia evolving since the age of 18. She was treated with risperidone in 2014 during a previous hospitalization and was subsequently lost to follow-up. In march 2019, she was hospitalized for an exacerbation of psychotic symptoms with disorganization, hallucination, delusion, and anxiety. Risperidone was reintroduced up to 4 mg/day together with loxapine 350 mg/day. Paliperidone injections were initiated with the first injection realized on April 1, 2019. Within a few days, the patient suffered from catatonic symptoms with stupor, partial mutism, fixed gaze, grimacing, echopraxia, waxy flexibility, ambitendency, associated with neurovegetative signs (125 bpm). With 1 mg of lorazepam, a transient improvement was observed (BFCRS decreased from 15/69 to 9/69). Oral antipsychotics were stopped progressively over 4 days and lorazepam was increased up to 7.5 mg/day without efficacy, together with poor tolerance (balance disorders). Complete blood count returned normal. ECT was decided but was not immediately available. Two tDCS sessions were performed in the meanwhile, resulting in a spectacular improvement of both of catatonic syndrome (BFCRS went from 17 to 11/69) and neurovegetative signs (heart rate decreased from 125 to 80). Given this improvement, tDCS sessions were continued instead of ECT. Overall, twenty sessions of tDCS were performed (twice a day for 11 consecutive weekdays) with further BFCRS improvement. After the 16th session, BFCRS scored at 9/69 (on April 19), meaning a 52% symptom reduction. Aripiprazole was then cautiously initiated, allowing discharge from the hospital.

Case 5 (2019)

Mrs. J was a 59-year-old female patient with intellectual disability, epilepsy, as well as schizophrenia evolving since 2003. Between March 2018 and April 2019 she was treated by olanzapine (30 mg/day) with good efficacy. She already suffered from several episodes of catatonia, most of the them caused by treatment discontinuations. These episodes were treated with olanzapine. In April 2019, she was hospitalized for an exacerbation of delusion and catatonic symptoms, with stupor, mutism, waxy flexibility, negativism and posturing. BFCRS score was 18/69. After a positive zolpidem test (partial improvement of stupor and mutism), olanzapine was stopped and lorazepam was titrated up to 10 mg/day on April 15. ECT was scheduled to begin on April 19. tDCS sessions were initiated in the meanwhile with the first session on April 16. After 2 tDCS sessions, BFCRS scored at 9/69, meaning a symptom reduction of 61%. The catatonic syndrome subsequently worsened despite the continuation of tDCS (BFCRS at 16 on April 26 after 5 sessions), leading us to initiate ECT sessions on May 3. Further improvement was observed with ECT. Risperidone was then cautiously introduced up to 2mg/day, allowing discharge from the hospital.

Case 6 (2019)

Mr. C was a 26-year-old male patient with autism spectrum disorder, schizophrenia, and chronic catatonic syndrome evolving since 2006. Between 2006 and 2015, he received several oral antipsychotics including risperidone, aripiprazole, amisulpride and haloperidol. Clozapine (up to 650 mg/day) associated to aripiprazole (30mg) were introduced in 2018, allowing partial improvement. However, the patient still suffered from chronic catatonic symptoms with agitation, staring, catalepsy, grimacing, echolalia, stereotypy, mannerisms, verbigeration, rigidity, waxy flexibility, ambitendency and perseveration. In January 2017, he had several seizures requiring adjunction of lamotrigine (up to 400 mg/day), then switched to valproate (up to 2000 mg/day) in August 2018. In July 2019, the patient was hospitalized in order to adjust his treatment. BFCRS was then at 24/69. ECT was discussed but we feared that the antiepileptic treatment would induce poor efficacy and tolerance of ECTs. Finally, a tDCS course was decided. Ten sessions were performed (twice a day for 5 consecutive weekdays) without immediate clinical remission (unfortunately, BFCRS was not rated after the 10 sessions) even if a soothing effect was observed during the sessions. The patient was discharged and went in holidays with his family who latter reported clinical improvement (less hallucinations, fewer episodes of stupor and reduced rigidity). Two months later, BFCRS was scored ate 17/60 (meaning a symptom reduction of 29%).

Case 7 (2019)

Mr. D was a 54-year-old male patient suffering from schizophrenia since 1995 associated with recurrent catatonic episodes. He received at least 5 different antipsychotics, including clozapine (up to 600 mg/day) which was poorly tolerated. On top of his medication, ECT was required for several catatonic episodes (4 courses of ECT between 2003 and 2010). In January 2010, a cardiac arrest occurred during an ECT session which leaded to the diagnosis of dilated cardiomyopathy and hence to the contra-indication of ECT. In August 2019, the patient was hospitalized for a new episode of catatonic syndrome evolving for 6 months. At arrival, he was treated by vortioxetine 20 mg/day and lorazepam 1 mg/day (all antipsychotics had been stopped because of cardiac failure) and was stuporous, in a rigid, silent position, with his gaze fixed towards the ground (BFCRS oscillating between 17 and 22/69). A zolpidem test was performed, with a clinical response after 30mg were administered (BFCRS went from 19 to 5). Two sessions of tDCS were performed on August 19, but patient refused to continue the course. Lorazepam was titrated up to 15mg/day on August 28 leading to a partial response on negativism and opposition. Given the severity of the remaining symptoms and ECT contra-indication, tDCS was again offered to the patient. The first session of this second course was performed on August 29. After 14 sessions of tDCS were performed (twice a day), BFCRS went from 21/69 (on August 19 ; before tDCS) to 8/69 (on September 11), meaning an overarll 64% symptom reduction. tDCS were continued for 20 more sessions (twice a day for 7 consecutive days) without further improvement and were then stopped. Three weeks after the last session, a relapse of catatonic symptoms was observed (BFCRS scored at 19/69 on October 14). tDCS was resumed with 56 sessions performed and resulting again in a partial improvement of catatonic symptoms (BFCRS of 12 on November 18). Finally, it was decided to start again ECT on December 11, with subsequent improvement.

Case 8 (2019)

Mrs. G is a 24-year-old female patient who suffered from several major depressive episodes between 2013 and 2019. In September 2019, she was hospitalized for a first manic episode with psychotic symptoms. Upon arrival, her current treatment (olanzapine and clomipramine) was stopped and switched to lithium 800 mg/day and aripiprazole 15 mg/day. A sedative treatment with chlorpromazine (up to 800mg/day) was co-prescribed. Manic symptoms gradually disappeared with the onset of severe depression. A few days later, the patient suddenly presented catatonic symptoms with agitation, posturing, echopraxia, stereotypy, mannerisms, verbigeration, rigidity, perseveration, ambitendency and excessive sweating. BFCRS score was 14/69. After a positive response-test to lorazepam 2.5 mg, antipsychotics were stopped and lorazepam was titrated up to 7.5 mg/day on October 18, leading to partial relief of catatonic syndrome. Given symptoms severity, including vegetative signs (profuse sudation and heart rate at 100 bpm), ECT was offered but refused by the patient and her family. tDCS was then initiated in association with lorazepam 7.5 mg/day. Fourteen sessions of tDCS were performed (twice a day for 7 consecutive weekdays) allowing a rapid reduction in catatonic symptoms. BFCRS went from 14/69 on October 16 (before tDCS) to 0/69 on November 14^t^, meaning an overall 100% symptom reduction.
